# A randomised trial into the effect of an isolated hip abductor strengthening programme and a functional motor control programme on knee kinematics and hip muscle strength

**DOI:** 10.1186/s12891-015-0563-9

**Published:** 2015-05-03

**Authors:** Kathryn Palmer, Clair Hebron, Jonathan M Williams

**Affiliations:** Faculty of Health Sciences, School of Health Professions, University of Brighton, 49 Darley Road, BN20 7UR Eastbourne, East Sussex UK; Faculty of Health and Social Sciences, Bournemouth University, Royal London House, Christchurch Road, BH1 3LT Bournemouth, Dorset UK

**Keywords:** Q-angle, Squat, Single leg landing, Muscle strength, Knee valgus

## Abstract

**Background:**

Dynamic knee valgus and internal femoral rotation are proposed to be contributory risk factors for patellofemoral pain and anterior cruciate ligament injuries. Multimodal interventions including hip abductor strengthening or functional motor control programmes have a positive impact of pain, however their effect on knee kinematics and muscle strength is less clear. The aim of this study was to examine the effect of isolated hip abductor strengthening and a functional motor control exercise on knee kinematics and hip abductor strength.

**Methods:**

This prospective, randomised, repeated measures design included 29 asymptomatic volunteers presenting with increase knee valgus and femoral internal rotation. Participants completed either isolated hip abductor strengthening or a functional motor control exercise for 5 weeks. Knee kinematics were measured using inertial sensors during 2 functional activities and hip abductor strength measured using a load cell during isometric hip abduction.

**Results:**

There were no significant differences in dynamic knee valgus and internal rotation following the isolated hip abductor or functional motor control intervention, and no significant differences between the groups for knee angles. Despite this, the actual magnitude of reduction in valgus was 10° and 5° for the functional motor control group and strengthening group respectively. The actual magnitude of reduction in internal rotation was 9° and 18° for the functional motor control group and strengthening group respectively. Therefore there was a tendency towards clinically significant improvements in knee kinematics in both exercise groups. A statistically significant improvement in hip abductor strength was evident for the functional motor control group (27% increase; p = 0.008) and strengthening group (35% increase; p = 0.009) with no significant difference between the groups being identified (p = 0.475).

**Conclusions:**

Isolated hip strengthening and functional motor control exercises resulted in non-statistically significant changes in knee kinematics, however there was a clear trend towards clinically meaningful reductions in valgus and internal rotation. Both groups demonstrated similar significant gains in hip abductor strength suggesting either approach could be used to strengthen the hip abductors.

## Background

Knee injury is one of the most common musculoskeletal complaints affecting 20% of military personnel [1]. In particular the incidence of patellofemoral pain syndrome (PFPS) has been reported at 8.7% for British Army recruits [2] and 9.7% for anterior cruciate ligament (ACL) injuries during military training [2,3]. The consequences of these injuries are significant, therefore the causes and contributing factors are an important consideration in the prevention of such injuries. The causes of these knee injuries are likely to be multifactoral, although abnormal knee kinematics and specifically dynamic knee valgus and internal rotation (DKVIR) has been widely postulated as a factor in the etiology of both PFPS and non-contact ACL injury [4-8].

One explanation for the increase DKVIR is weakness in the hip abductors, as these muscles work to control femoral adduction and internal rotation [7,9]. Consequently, it has been suggested that strengthening these muscles may reduce the DKVIR [10]. However an alternative mechanism has been proposed to explain excessive DKVIR. Poor functional motor control (FMC) where altered recruitment of hip musculature is observed, has also been suggested to contribute to increased DKVIR [11,12]. This aberrant recruitment suggests that the overall motor strategy and movement patterns are ‘faulty’ resulting in excessive DKVIR. Therefore it is logical to suggest that if an aberrant functional motor control strategy is associated with excessive DKVIR and risk of PFPS then targeting this with a specific intervention should result in an alteration of DKVIR.

Previous studies have demonstrated that improvements in pain and function in PFPS and a reduction of non-contact ACL risk can be achieved through programs targeting both hip abductor strengthening and functional motor control [9,13-16]. There is however conflicting evidence that such programmes result in a change in DKVIR, as some demonstrate changes in DKVIR [16] while others failed to result in change of DKVIR [9,17,18]. This conflicting evidence could be explained by differences in baseline DKVIR values, as the presence of increased DKVIR was often not part of the inclusion criteria or by the multimodal and non-homogeneous exercises employed. Additionally, despite these studies suggesting that altering the DKVIR may be possible, such multimodal programs fail to provide adequate insight into the mechanism behind any alterations observed. Furthermore previous studies did not investigate multiple time points, preferring pre-and post-testing only which fails to describe changes with respect to time.

The effect of isolated hip strengthening or functional motor control exercise on DKVIR has not been adequately established. Such insights will be useful for injury prevention programs and will improve our understanding of the mechanisms behind changes in DKVIR. The aim of this study was to investigate the effect of two commonly used clinical approaches to alter DKVIR: isolated hip abductor strengthening and a FMC exercise, on pain free military participants identified as having increased DKVIR at baseline.

## Methods

A prospective, randomised, experimental repeated measures design was used. Forty-two military personnel volunteers were recruited from four military bases to participate in the study. Volunteers were initially visually screened by the lead investigator (KP), a Physiotherapist with extensive post-graduate training, to determine the presence of increased DKVIR. Two-dimensional frontal plane analysis has been recommended as a screening tool for the presence of increased DKVIR and has been validated compared with three-dimensional motion analysis during single leg squatting [19]. Exclusion criteria included a history of knee pain in the proceeding twelve months, history of surgery, known rheumatologic, neurological or cardiovascular disorders with raised blood pressure. Participants were assigned to one of two groups using stratified randomisation ensuring equal distribution of females among the groups, as gender has been identified as a confounding variable with females often displaying greater DKVIR angles [20,21]. Demographic data for the two groups are presented in Table 1.

**Table 1:** **Demographic characteristics of participants (mean (SD))**

	**Abd Str (n = 15)**	**Mot Con (n =14)**
Male/female	11/4	10/5
Age (years)	30.3 (8.8)	29.6 (9.7)
Height (m)	1.8 (0.1)	1.7 (0.1)
Weight (kg)	80.5 (12.7)	69.3 (11.2)
BMI (kg/m^2^)	30.3 (3.1)	23.3 (3.2)

(Abd Str; abductor strengthening group, Mot Con; functional motor control group).

This study sought to determine a change in knee valgus for greater than 3° thought to represent a clinically meaningful change and representative of earlier studies [9,21]. Along with a standard deviation value determined from Earl and Hoch [9] and power set at 80% and alpha at 0.05, the required sample size of 16 per group was determined. Over recruitment of 30% was estimated to manage drop outs.

**Figure 1:**
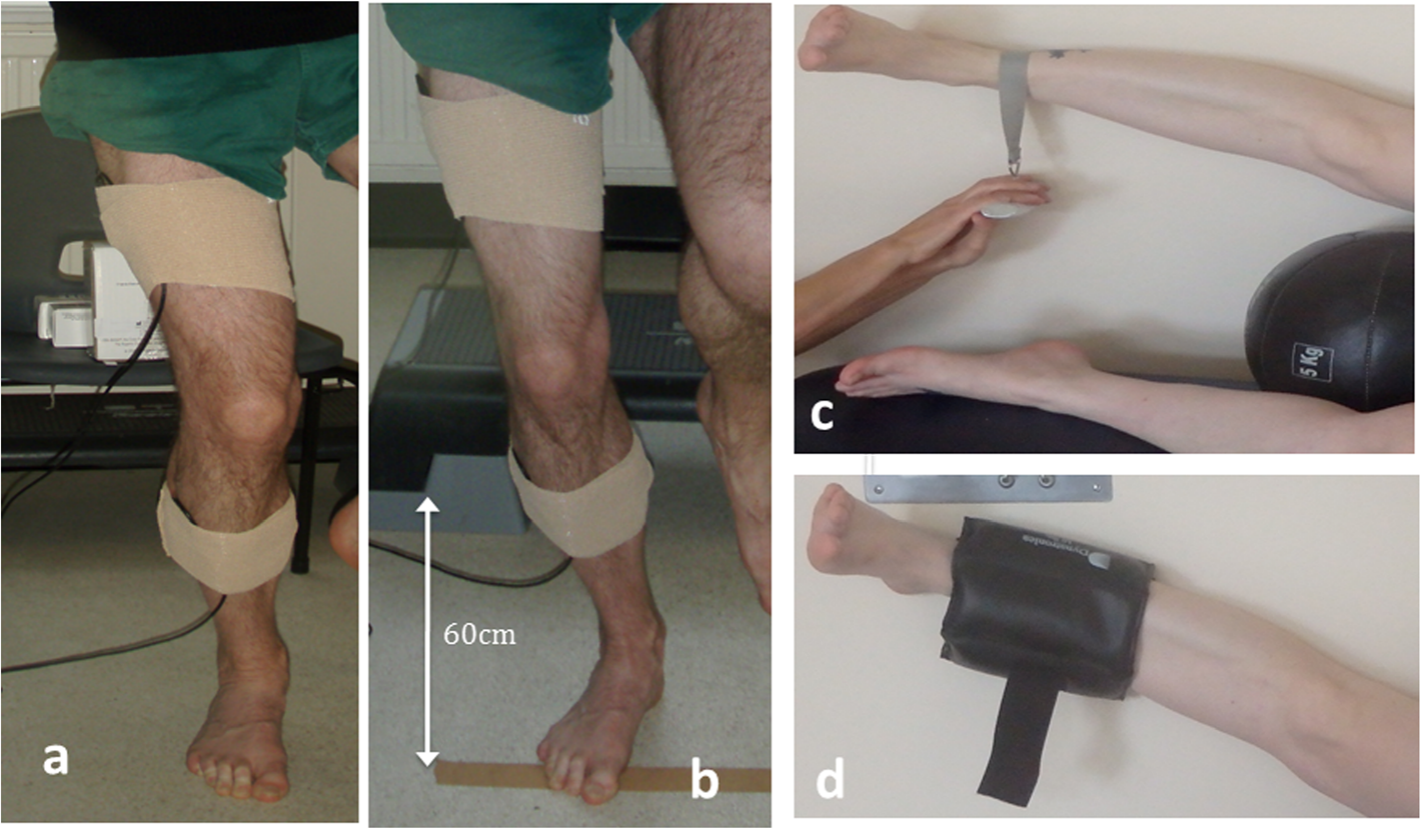
Sensor attachment, strength testing and strengthening exercise. **a**, Single leg squat; **b**, Single leg landing; **c**, Hip abductor strength testing; **d**, Hip abductor strengthening exercise.

Participants gave written informed consent prior to taking part in the study following explanation of procedures and risks. Additional consent was granted from one individual to include their photo in this publication demonstrating the experimental set-up (Figure 1). Ethical approval was granted by the Faculty of Health and Social Science Research Ethics and Governance Committee at the University of Brighton and the Ministry of Defence Research Ethics Committee (reference number; 346/GEN/12).

### Procedure

All testing was conducted in shorts and barefoot to prevent any potential variations that may have occurred due to footwear. DKVIR were measured during single leg squatting and single leg landing using two inertial sensors with a custom built datalogger and software (ThetaMetrix; Waterlooville, UK). Data were collected at a sampling rate of 100Hz. Accuracy of these sensors has been reported by the company to be 0.5 degrees and reliability measurements (ICC) for human studies reported as 0.71-0.99 [22]. One sensor was placed on the lateral thigh (midway on a line connecting the lateral femoral epicondyle and greater trochanter) and the other over the lateral lower limb (midway along a line connecting the lateral femoral epicondyle and the lateral malleolus) with the knee straight. Sensors were attached using double-sided tape and reinforced using elastic wrap to minimize soft tissue artifact (Figure 1a and b).

Participants performed a single leg squat on their testing leg, squatting until their upper posterior thigh came into contact with a stool (height 550 mm). The rate of the single leg squat was controlled to one-second descent and one second ascent to minimize the effect of velocity on kinematics and participants were instructed to maintain an upright trunk (Figure 1a). Participants performed a double leg jump from a step box (200 mm) with a single leg landing to land on a target 600 mm from the step box. Upon landing, participants were asked to ‘stick the landing’ and maintain single leg balance for one second after each trial (Figure 1b). Failure to meet these criteria resulted in negation and repetition of that trial. A short familiarisation period preceded the testing. Both tasks were completed three times.

Hip abduction strength was measured using maximal force production (in kg) measured by a load cell (Duronic, UK). Strength testing was performed with participants’ side lying on a treatment plinth, their back against the wall. The underneath leg was flexed at the hip and knee with their top leg (testing leg) straight, in contact with the wall and resting on a medicine ball (to standardize the hip abduction angle). Participants were asked to lift their leg as forcefully as possible maintaining heel contact with the wall for three seconds with maximal force being measured. Three experimental trials with a 30 second rest between trials were performed (Figure 1c).

Testing of both knee kinematics and hip strength was completed at weekly intervals for five weeks.

#### Isolated hip abductor strengthening group

The hip abductor strengthening protocol was based on evidence from the American College of Sports Medicine [23]. Initial strength was determined and a load of 80% of this strength was provided in the form of ankle weights. Side lying hip abduction with ankle weights was performed until failure with each participant completing 3 sets, every other day. The load was adjusted at each testing session based on the strength testing results. The side lying position was used as it has been shown to create the greatest activation of the hip abductors, has been shown to result in strength gains and reflects common clinical practice [16,24,25] (Figure 1d). All strengthening was completed unsupervised, with participants required to complete training diaries.

#### Functional motor control group

The FMC exercise specifically aimed to target the cognitive and then automatic control of knee kinematics, a method common in clinical practice [16,17,26]. This method sequentially challenges the control of the knee position during squatting by gradually progressing from double leg to single leg squatting. Initial assessment determined how much support was required from the non-exercising leg in order to main the desired knee alignment. The following is a guide to the progression:

- Opposite foot on low step and hand support
- Opposite foot on low step, no hand support
- Opposite lower leg against the wall
- Opposite foot against the wall
- Just hand support
- No support

Once established, participants were instructed to work on squatting, maintaining the desired alignment for up to 25 repetitions providing alignment could be maintained. This was practiced daily and progressed weekly using a diary to record adherence.

Kinematic data were processed in Matlab (Mathworks, R2008b). Each sensor provides absolute orientation in the form of Euler angles and from these, relative angles between the sensors were calculated from the direction cosine matrices to give flexion/extension, rotation and valgus/varus angles. Valgus was defined as negative as was internal rotation. A specific algorithm was developed to mathematically reorientate the sensors to offset for the slight differences in initial orientation associated with mounting the sensors each week.

### Statistical analysis

The maximum knee angles were identified for each of the three trials and repeated measures reliability statistics calculated to determine the consistency of the kinematic variables (intraclass correlation coefficient (ICC (consistency), standard error of measurement (SEM) and minimal detectable change (MDC)). The mean of the three trials for each dependent variable were calculated during single leg squat and single leg landing. Mean maximum force from the three trials were determined and also explored for repeated measure reliability (ICC (consistency), SEM, MDC). Mean kinematics and force were tested for normality using the Shapiro-Wilk test and all data were normally distributed. The effects of the exercises were determined using one-way repeated measures ANOVA, having first checked for sphericity using Mauchly’s test. The model was created using time as the within subject factor with 6 levels (week 0 to 5). Between group comparisons were made using a multivariate ANOVA with dependent variables of knee kinematics and strength and the fixed factor group. Post-hoc testing was completed where appropriate. A comparison between groups for frequency of responder was completed using the Chi-squared test, where responder was determined as an individual who decreased their valgus or internal rotation by 5 degrees or more. All statistical analysis was completed using statistical package for social science (SPSS) software (version 20, IBM). Significance levels were p < 0.05.

## Results

**Figure 2:**
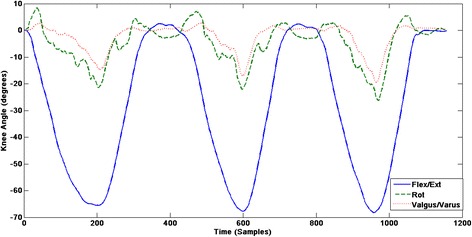
A single participant’s knee kinematics during single leg squatting.

**Figure 3:**
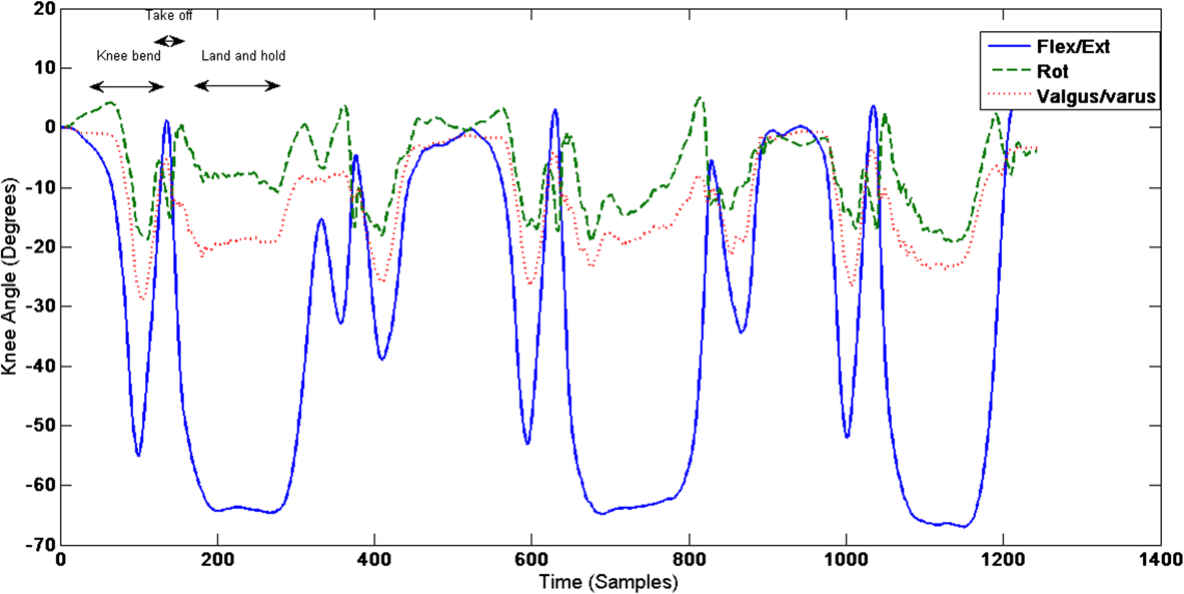
A single participant’s knee kinematics during single leg landing.

A single participant’s kinematic data for squatting and landing are presented in Figures 2 and 3. Reliability of repeated measurements were excellent for all dependent variables with small SEM and MDC values (Table 2) demonstrating good consistency in movement patterns and strength testing. Single leg squat and single leg landing data across the six time points are presented in Table 3.

**Table 2:** **Repeated measures reliability of measurements**

	**Knee Angle Kinematics**				**Hip Abductor Strength**	
	**Abd Str**		**Mot Con**		**Abd Str**	**Mot Con**
	**Valgus**	**Rotation**	**Valgus**	**Rotation**	**Force/kg**	**Force/kg**
ICC (3,1)	0.98	0.97	0.96	0.96	0.97	0.97
SEM (deg)	3.8	4.2	3.3	3.8	1.4	1.0
MDC (deg)	5.3	5.7	5.0	5.4	3.3	2.8

ICC; intraclass correlation coefficient, SEM; Standard error of measurement, MDC; minimal detectable change. Abd Str; abductor strengthening group, Mot Con; functional motor control group, deg; degrees.

**Table 3:** **Mean (SD) knee angle data (degrees)**

	**Week 0**			**Week 1**			**Week 2**			**Week 3**			**Week 4**			**Week 5**		
	**Flex**	**Rot**	**Val**	**Flex**	**Rot**	**Val**	**Flex**	**Rot**	**Val**	**Flex**	**Rot**	**Val**	**Flex**	**Rot**	**Val**	**Flex**	**Rot**	**Val**
SQUAT																		
AbdStr	58.7 (8.1)	-15.2 (22.5)	-19.4 (19.8)	59.6 (9.0)	3.0 (26.5)	-14.8 (25.4)	61.1 (7.8)	-0.2 (22.0)	-14.4 (21.1)	59.3 (8.8)	5.7 (23.9)	-12.1 (22.0)	61.5 (9.4)	-1.6 (24.8)	-8.1 (25.5)	62.9 (8.8)	2.6 (27.2)	-13.8 (23.9)
MotCon	58.0 (8.1)	-16.2 (17.0)	-16.2 (10.2)	55.0 (7.6)	-15.3 (8.5)	-11.7 (8.2)	57.1 (10.3)	-10.4 (16.9)	-12.3 (11.2)	53.6 (8.1)	-11.9 (17.8)	-12.7 (17.9)	51.7 (7.5)	-13.5 (16.9)	-16.3 (17.3)	57.1 (7.6)	-7.1 (13.4)	-6.2 (19.8)
LAND																		
AbdStr	51.9 (10.0)	-10.8 (11.8)	-9.8 (11.9)	58.1 (9.4)	-9.8 (10.7)	-9.9 (9.0)	54.7 (10.1)	-13.6 (13.1)	-14.2 (16.0)	58.9 (11.7)	-7.3 (9.3)	-6.3 (9.7)	53.7 (10.4)	-4.5 (17.2)	-9.6 (15.8)	56.6 (11.7)	-6.5 (11.3)	-8.9 (10.6)
MotCon	48.1 (8.5)	-5.2 (9.2)	-5.2 (9.6)	45.7 (9.3)	-5.1 (8.2)	-8.9 (9.8)	49.5 (9.4)	-4.5 (11.1)	-3.8 (11.7)	50.9 (9.0)	-4.2 (8.7)	-3.1 (11.4)	47.8 (8.8)	-5.6 (10.6)	-6.2 (13.8)	48.8 (10.4)	-4.8 (10.2)	-3.6 (12.3)

Flex, Flexion; Rot, Rotation; Val, Valgus; AbdStr, Abductor Strengthening group; MotCon, Functional Motor Control group; Squat, single leg squat; land, single leg land.

The functional motor control exercise group demonstrated no significant difference in knee valgus or rotation across time for single leg squat (valgus p = 0.124; rotation p = 0.096) or single leg landing (valgus p = 0.182; rotation p = 0.361). The isolated hip abductor strengthening group demonstrated no significant difference in valgus or rotation across time for single leg squat (valgus p = 0.614; rotation p = 0.614) or single leg landing (valgus p = 0.546; rotation p = 0.785).

**Figure 4:**
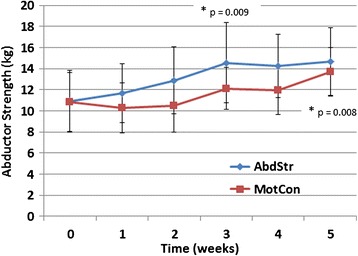
Mean abductor strength (kg) for both groups. Error bar represents one standard deviation.

A significant difference was identified for strength across time in the strengthening group (p = 0.009) and the functional motor control group (p = 0.008). Differences between week 0 and 1 were evident along with weeks 4 and 5 for the strengthening group and between weeks 0, 1, 2 and week 5 for the functional motor control group (Figure 4).

Multivariate ANOVA testing revealed no significant difference between groups for knee angles during squatting (p = 0.551) or single leg landing (p = 0.450). Furthermore no between group differences were identified for strengthening effect (p = 0.475).

There were four responders in the strengthening group, compared to six for the motor control group for rotation along with six responders in the strengthening group compared to nine for the motor control group for valgus. Chi-squared testing revealed no significant difference in the number of responders between the two groups for valgus (p = 0.247) or rotation (p = 0.397).

## Discussion

The current study investigated the effect of individual isolated exercises on knee kinematics during single leg squatting and landing, as well as hip abductor strength, in those who present with visually abnormal dynamic knee angles. The results demonstrate that neither group demonstrated a statistically significant difference in knee kinematics during the five weeks of exercise. There were significant differences for both groups in the development of strength of the hip abductors demonstrating both exercises were effective in improving hip abductor strength. Despite this no between group differences were established suggesting neither group was more effective at altering kinematics or muscle strength.

The results of this study show that over a five week period both isolated hip muscle strengthening or a functional motor control exercise failed to significantly alter knee kinematics. These findings are in agreement with the majority of the literature, including in those with knee pain [9] and healthy individuals [17,18]. Previous studies employed multimodal exercise programmes consisting of hip strengthening ranging from 3 to more than 10 exercises. Therefore the current study adds to the knowledge by demonstrating that specific isolated exercise also results in no statistically significant change in knee kinematics. These findings suggest the alteration of kinematic profiles during squatting and landing may be inherently complex and that the positive benefits attributed to these exercises in PFPS may be due to mechanisms unrelated to the kinematics measured in the current study. Indeed studies observing clinical benefits seem to report on changes in moments rather than kinematics [9,27].

Despite the lack of statistical significance there was a trend towards decreased valgus and internal rotation over time for both groups. The functional motor control group demonstrated a mean decrease of 10° and 9° for valgus and internal rotation respectively along with a 5° and 18° respective change for valgus and internal rotation in the strengthening group. Previous literature has attempted to identify a meaningful change in knee valgus leading to the suggestion that 3° may be sufficient in determining clinically meaningful change [21]. The magnitude of change evident in the current study therefore is likely to represent a clinical meaningful change and is in accordance with previous studies investigating the effects of multimodal exercises on knee valgus [16]. Moreover the resultant valgus magnitudes, as measured at the beginning and end of the study, demonstrate a shift from a high risk category to a low risk category for established valgus angle population norms [28].

The current study demonstrates that there appears to be a large amount of variability in the response to the exercises within the two groups, suggesting the response is not homogenous. Despite this a group comparison investigating the frequency of responders failed to demonstrate a significant difference between the functional motor control group and the strengthening group. This suggests that individuals were just a likely to reduce their valgus or internal rotation with either exercise. A five degree change was utilized as the definition of responder due to the minimal detectable change values produced using the above described measurement technique. It is not clear from the results of this study why some individuals responded whereas some did not, and this could form a basis for future studies.

It is interesting to note that the magnitude of change in valgus, within the FMC group, was double that of the strengthening group. Moreover the magnitude of change in internal rotation, within the strengthening group, was double that of the FMC group. This seems to suggest that the FMC exercise might be better at altering knee valgus. The may be due to the lack of specific instructions regarding femoral rotation during the FMC exercise, where instructions focusing more on overall knee alignment were used. However it could be suggestive that this exercise approach is better suited at addressing knee valgus, when knee valgus is the dominant impairment. The opposite may be true for hip abductor strengthening. This approach seemed to yield superior results for internal rotation. These results suggest that it may be possible for clinicians classify individuals based on their primary impairment (valgus or internal rotation) and design specific targeted interventions based on the findings of the current study. However it is worthy to note that these changes in kinematics were trends and therefore this concept requires further scientific study.

Assessing knee kinematics during the single leg landing task was important as it is likely to represent a more subconscious and automatic task, and more akin to the demands of many military tasks. There were no statistically significant differences in knee valgus and internal rotation for the single leg-landing task for either group. Moreover the actual magnitude of change was small. Despite this, an interesting pattern of movement was observed. Valgus and internal rotation were evident when the participants flexed their knee prior to take off, but often on landing minimal valgus and rotation was observed (Figure 3). It is possible that the anticipation or recognition of the relatively higher loading task triggered movement patterns which differed to those associated with the knee flexion prior to take off.

There was a significant increase in hip abductor strength following both exercises, with a mean increase of 35% (3.8 kg) for the strengthening group and 27% (2.9 kg) for the functional motor control group. Neither group resulted in significantly greater strength gains. Previous studies have demonstrated slightly less mean percentage change following targeted hip abductor strengthening interventions [12] and multimodal exercise regimens (from 10% to 28%) [9,16,18]. The adherence to the ACSM guidelines may explain the slighter greater gains evident in the strengthening group when compared to the literature [23]. To the authors knowledge this is the first study to assess the effect of a functional motor control exercise on hip abductor strength, demonstrating statistically significant improvements. This novel finding suggests that exercises aimed at improving functional motor control are likely to result in concomitant strength improvements of a significant magnitude. In analysing the functional motor control exercise reasons for the strengthening effect can be hypothesised. The single leg squatting requires hip abductor muscle activation on the weight bearing side in order to control pelvis positioning in the frontal plane. Electromyography has demonstrated that single leg squatting can result in an 82% maximum voluntary contraction of gluteus medius [29]. This suggests that the functional motor control exercise may have provided enough stimuli to result in overload and strength gains in the hip abductor muscles. This finding questions the need to use isolated strengthening of the hip musculature in individuals with increased knee valgus and internal rotation.

Limitations of the current study should be acknowledged. This study was conducted using pain-free individuals and therefore caution is advised if attempting to extrapolate the results to those with knee pain. It is not possible to know if those with knee pain would respond in the same way to those in this study. No control group was used as it was deemed unnecessary because our aim was to explore the effect of isolated approaches to altering knee angles rather than test an ‘intervention.’ It is possible that alterations in dependent variables were due to other factors such as natural variation over time, measurement variability and not to the independent variables used in this study. The sample size was small. Despite a sample size calculation being conducted using previously published data, the required sample size was not quite achieved due to a larger than expected drop out rate. However, a post-hoc power calculation did determine that the current study had a power of 0.89 based on a pooled standard deviation and mean change in valgus angle. The standard deviations in the current study were large reflecting the natural variability across this population for the tasks investigated. It is likely that such deviations will have affected the statistics and future studies should perhaps investigate a more homogenous task or population. The duration of the study was short as it is known that strength changes continue for longer than 5 weeks therefore it is unlikely a strengthening ceiling was reached within just 5 weeks [30,31]. Despite strength changes being evident in the current study, it is not clear whether a longer study duration would have influenced the findings.

## Conclusion

The results from this study demonstrate that there were no statistically significant differences in knee kinematics following 5 weeks of an isolated hip abductor strengthening programme or a FMC exercise programme. However, there was a tendency towards clinically significant improvements in knee kinematics for both groups resulting in a reduction in dynamic knee valgus and internal rotation. Both groups demonstrated significant and similar gains in strength of the hip abductors. It is possible that such approaches could lead to a reduction of knee valgus and internal rotation and subsequent reduction in knee pain risk. Further studies should evaluate the ability of such approaches to alter knee kinematics long term with simultaneous monitoring knee injury risk.

## Abbreviations

PFPS: Patellofemoral pain syndrome
ACL: Anterior cruciate ligament
DKVIR: Dynamic knee valgus and internal rotation
FMC: Functional motor control exercise
ICC: Intraclass correlation coefficient
SEM: Standard error of measurement
MDC: Minimal detectable change
mm: Millimeters
kg: Kilogram
ANOVA: Analysis of variance
SPSS: Statistical package for the social sciences
ACSM: American College of Sports Medicine
Abd Str: Abductor strengthening group
Mot Con: Functional motor control group
